# Oral Administration of Fermented Papaya (FPP^®^) Controls the Growth of a Murine Melanoma through the In Vivo Induction of a Natural Antioxidant Response

**DOI:** 10.3390/cancers11010118

**Published:** 2019-01-20

**Authors:** Mariantonia Logozzi, Davide Mizzoni, Rossella Di Raimo, Daniele Macchia, Massimo Spada, Stefano Fais

**Affiliations:** 1 Department of Oncology and Molecular Medicine, Istituto Superiore di Sanità, Viale Regina Elena 299, 00161 Rome, Italy; mariantonia.logozzi@iss.it (M.L.); davide.mizzoni@iss.it (D.M.); rossella.diraimo@iss.it (R.D.R.); 2 Centro Nazionale Sperimentazione e Benessere Animale, Istituto Superiore di Sanità, Viale Regina Elena 299, 00161 Rome, Italy; daniele.macchia@iss.it (D.M.); massimo.spada@iss.it (M.S.)

**Keywords:** FPP^®^, tumor, prevention, adjuvant treatment, anti-oxidant effect

## Abstract

Prolonged oxidative stress may play a key role in tumor development. Antioxidant molecules are contained in many foods and seem to have a potential role in future anti-tumor strategies. Among the natural antioxidants the beneficial effect of Fermented Papaya (FPP^®^) is well known. The aim of this study was to investigate the effects of orally administered FPP^®^ in either the prevention or treatment of a murine model of melanoma. The tumor growth was analyzed together with the blood levels of both oxidants (ROS) and anti-oxidants (SOD-1 and GSH). The results showed that FPP^®^ controlled tumor growth, reducing the tumor mass of about three to seven times vs. untreated mice. The most significant effect was obtained with sublingual administration of FPP^®^ close to the inoculation of melanoma. At the time of the sacrifice none of mice treated with FPP^®^ had metastases and the subcutaneous tumors were significantly smaller and amelanotic, compared to untreated mice. Moreover, the FPP^®^ anti-tumor effect was consistent with the decrease of total ROS levels and the increase in the blood levels of GSH and SOD-1. This study shows that a potent anti-oxidant treatment through FPP^®^ may contribute to both preventing and inhibiting tumors growth.

## 1. Introduction

Tumors are in fact a multifactorial pathology in which, in addition to environmental factors, some phenotypes common to all malignant tumors have been shown to have a role in tumor development and growth. These include inflammation, oxidative stress, hypoxia and microenvironmental acidity [1,2,3,4,5,6,7,8,9,10,11]. Recent evidence suggests that these factors induce the development of a very hostile micro-environment leading to a progressive selection of cells armed to survive in a hypoxic, acidic and generally toxic microenvironment [12]. This has triggered the so-called “micro-evolutionary” hypothesis predicting that the tumor environment induces a sort of selection of cells able to survive in conditions that are extremely hostile for normal cells [12,13,14,15]. Hence the micro-evolutionary process does not modify existing cells but selects the most suitable for the tumor microenvironment: therefore a malignant cell is not a normal cell that is transformed into a tumor cell through an accumulation of mutations, but the most suitable cell to survive in the tumor microenvironment [12].

Our research group has helped to demonstrate that the tumor microenvironment is typically acidic [5,16,17,18] and tumor acidosis has a key role in the proliferation, metastatic behavior and resistance to therapy [5,19]. However, it is now clear that prolonged oxidative stress may play a key role in predicting tumor growth [3,9,11]. The role of chronic inflammation in promoting tumor development and progression has long been known.

However, our study focused on the possibility of interfering with the initial accumulation of oxidative molecules and on the possibility of verifying the effect of oxidative molecules on tumor growth. In fact, the rapid turn-over of tumor cells leads to the accumulation of a large amount of ROS resulting in oxidative stress [20,21,22]. Oxidative stress, by weakening the endogenous antioxidant system and inducing cellular damage to proteins, lipids, carbohydrates and DNA [23], is therefore indicated as responsible or partly responsible for numerous diseases including cancer [11,24,25].

The reduction of oxidative stress and therefore of the redox balance alteration are necessary conditions for the restoration of normal cellular functions. However, most of the current strategies in the treatment of tumors based mainly on surgery, chemotherapy and radiation therapy are unable or insufficient to achieve this goal because they result in numerous side effects, paradoxically due above all to the induction of oxidative stress and consequent cell damage [26]. Cancer is a problem that now has a global reach and unfortunately it is very far from being solved. In fact, according to the latest estimates provided by the International Agency for Research on Cancer (IARC), both cancer incidence and mortality have increased in 2018 [27]. Prevention has therefore become a central topic in the fight against cancer, and at the core of prevention nutrition plays a major role. A diet based on fruits and vegetables, also recommended by the “World Health Organization” and the “World Cancer Research Fund”, seems to be able to contribute substantially to preventing the onset of most cancers [28,29,30,31,32,33]. The presence of antioxidants (such as lycopene, beta carotene, polyphenols, anthocyanins) seems to be one of the key factors in the prevention effect of the diet [28,29,30,31]. Among the natural antioxidants with immunostimulating and immunomodulating activities, the beneficial effect of Fermented Papaya (FPP^®^) (Immun’Âge^®^) is known [34,35,36,37].

Papaya is the fruit of the *Carica papaya* Linn, native to Central America (southern Mexico) and South America and nowadays grown in most tropical countries. Traditionally used as a medicinal fruit, *Carica papaya* Linn is rich in polyphenols and has antioxidant [38,39], immunostimulants [37,40] anti-inflammatory [41] properties and also beneficial effects in wound healing [42,43,44].

The presence of beneficial compounds with anticancer properties known as α-tocopherol [45], flavonoids [46], benzyl isothiocyanate [47,48] and lycopene [49] also give this fruit anti-tumor capacity [50,51]. These properties are more represented in Fermented Papaya (FPP^®^), a natural functional food (nutraceutical) able to support the natural antioxidant systems of the organism, produced with a technologically advanced and patented process, registered by the Japanese Patent Office on September 14th 2018 as ATP production promoter, mitochondrial activity promoter, and immunostimulant (Patent Number: 6401792) [37]. This process lasts 10 months and seems to increase the properties of “fresh” papaya, especially its antioxidant and digestive capacities, by concentrating nutritional compounds (such as carbohydrates, vitamins, minerals, lycopene) and by increasing proteolytic enzymes such as papain, which catalyzes the hydrolysis of proteins to oligopeptides [35]. In addition, the typical intake of FPP^®^ sublingually allows to achieve better results than the fresh fruit with a lower dose; in fact this route of administration proves to be faster and more effective, not going through the digestive system and the hepatic metabolism.

The presence of beneficial compounds with anticancer properties known as α-tocopherol [45], flavonoids [46], benzyl isothiocyanate [47,48] and lycopene [49] also give this fruit anti-tumor capacity [50,51]. These properties are more represented in Fermented Papaya (FPP^®^), a natural functional food (nutraceutical) able to support the natural antioxidant systems of the organism, produced with a technologically advanced and patented process, registered by the Japanese Patent Office on September 14th 2018 as ATP production promoter, mitochondrial activity promoter, and immunostimulant (Patent Number: 6401792) [37]. This process lasts 10 months and seems to increase the properties of “fresh” papaya, especially its antioxidant and digestive capacities, by concentrating nutritional compounds (such as carbohydrates, vitamins, minerals, lycopene) and by increasing proteolytic enzymes such as papain, which catalyzes the hydrolysis of proteins to oligopeptides [35]. In addition, the typical intake of FPP^®^ sublingually allows to achieve better results than the fresh fruit with a lower dose; in fact this route of administration proves to be faster and more effective, not going through the digestive system and the hepatic metabolism.

According to recent studies, FPP^®^ has proved to be an excellent antioxidant and an excellent nutraceutical adjuvant in combined therapies against several diseases, including tumors [36,37,38,51,52,53]. Actually, FPP^®^ is not simply a free radical neutralizer, but a free radical regulator [54] and an immunomodulator [42,43,55,56,57]. There is some experimental evidence on the mechanisms by which FPP^®^ interferes with the production and accumulation of free radicals even if there is much more to be verified [58,59].

Clinical studies and studies on experimental animals have shown that FPP^®^ can have an anti-aging effect [60] but also can contribute to fight against diseases in which oxidative stress plays a pathological role, such as neurodegenerative ones, hypertension and diabetes mellitus, but also diseases of the gastrointestinal tract [38,40,53,54,61,62].

In the present study the effects of orally administered FPP^®^ in the prevention and treatment of melanoma were investigated, using an immunocompetent mouse model (C57/BL) inoculated with B16 melanoma cells. We have identified the optimal treatment conditions with FPP^®^ with the final goal of evaluating its ability to keep tumor size under control and to study how oxidative stress directly affects tumor growth. So we measured the levels of oxidants (total ROS) and antioxidants (GSH and SOD-1) in mice’s blood in relation to the size of the tumor in order to provide the proof of concept that FPP^®^ may counteract tumor growth through inhibition of oxidative molecules production and accumulation, thus contributing to restore a redox balance in the whole body.

## 2. Results

### 2.1. Growth Curve of B16F10 in C57/BL Mice

**Figure 1:**
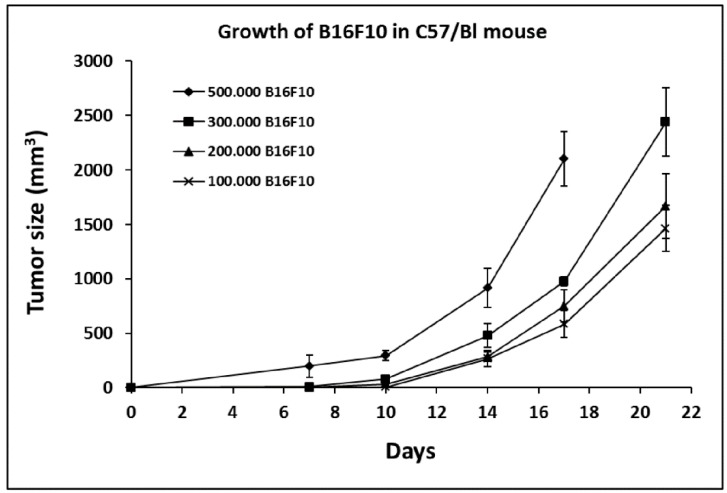
Growth of B16F10 in C57/BL mice. Growth curve of B16F10 in C57/BL mice inoculated with an increasing number of B16F10 cells, respectively 100,000, 200,000, 300,000 and 500,000 cells. C57/BL mice were divided into four groups and each group consisted of five animals for statistical significance. Data are expressed as means ± SE. Tumor size is expressed in mm^3^.

In order to identify the optimal conditions for treatment with FPP^®^ and to evaluate specific effects, C57/BL mice were inoculated with an increasing number of B16F10 cells, starting from 100,000 cells up to 500,000 cells. Figure 1 shows that inoculation 500,000 cells/mouse led to a very rapid tumor growth a condition in no way suitable for evaluating the FPP^®^ antitumor effect (tumor mass reaching a size of 197 ± 104 mm^3^ and up to 2101 ± 250 mm^3^ after 17 days). We thus used the model of 300,000 cells inoculation for the dose-response studies and the either 200,000 or 100,000 cells inoculations for studies on the therapeutic and the preventive effect of FPP^®^ (Figure 1).

### 2.2. Dose-Response Curve in C57/Bl Mice Treated with FPP^®^ by Oral Gavage

**Figure 2:**
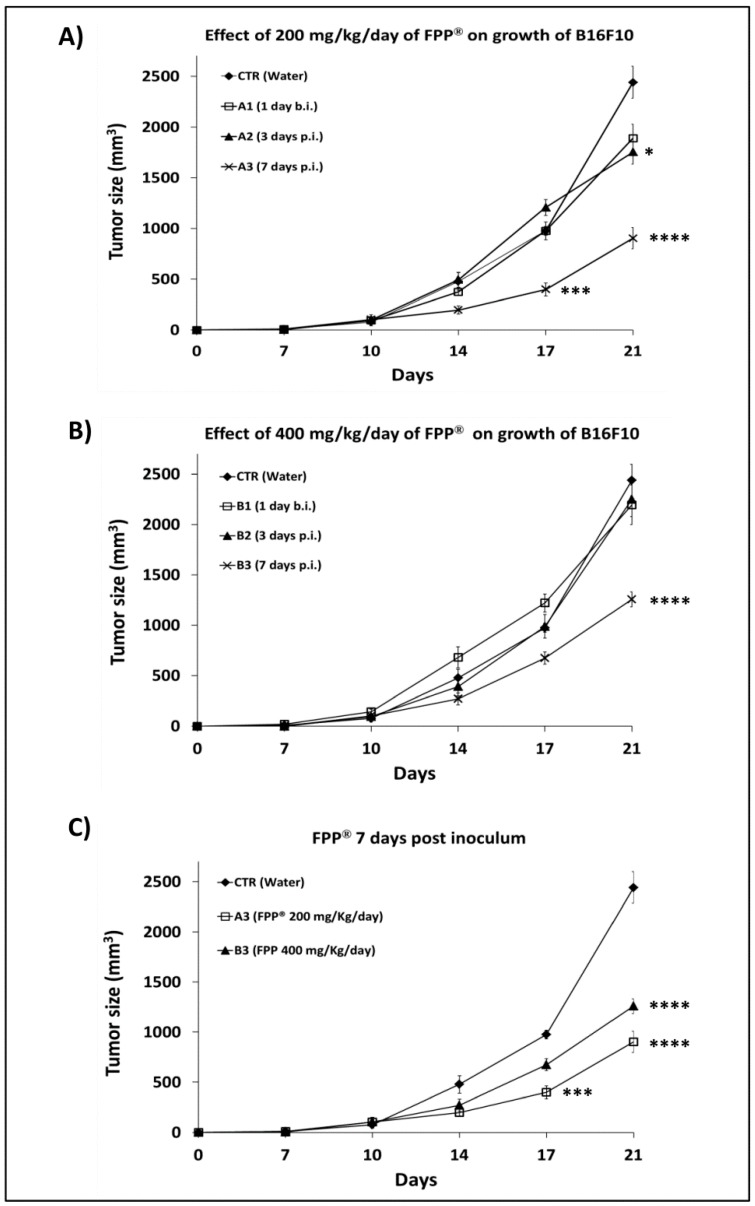
Effect of 200 mg/Kg/day and 400 mg/Kg/day of FPP^®^ through oral gavage on growth of B16F10 in C57/BL mice. Dose-response curve of FPP® on tumor growth (mm^3^) in C57/BL mice treated by oral gavage with 200 mg/Kg (group A1, A2, A3) and 400 mg/Kg of FPP^®^ (groups B1, B2, B3) every day without interruption until animal sacrifice, after 21 days from the inoculum of B16F10 cells (300000). Mice were divided into seven groups of five animals (control, A1, A2, A3, B1, B2, B3) and were treated with FPP^®^ respectively 1 day before the inoculation of the cells (groups A1, B1), 3 (groups A2, B2) and 7 (groups A3, B3) days after the inoculation of the cells. Control group is the untreated group. (**A**) Effect of 200 mg/Kg/day of FPP^®^ on growth of B16F10 in C57/BL mice (groups A1, A2, A3) until animal sacrifice. (**B**) Effect of 400 mg/Kg/day of FPP^®^ on growth of B16F10 in C57/BL mice (groups B1, B2, B3) until animal sacrifice. (**C**) Effect of FPP^®^ treatment 7 days post inoculum of the cells on growth of B16F10 in C57/BL mice comparing treatment with 200 mg/Kg/day of FPP^®^ (group A3) and 400 mg/Kg/day of FPP^®^ (group B3). Data are expressed as means ± SE. * *p* < 0.05, *** *p* < 0.0005, **** *p* < 0.0001.

To obtain the dose-response curve of FPP^®^ on tumor growth in C57/BL mice, 300,000 B16F10 cells were inoculated s.c. into each mouse and the mice treated with FPP^®^ every day without interruption until animal sacrifice. The mice were divided into seven groups of five animals and treated by oral gavage with FPP^®^ at the concentration of either 200 mg/Kg/mouse (Figure 2A) or 400 mg/Kg/mouse (Figure 2B). Moreover, the two different doses were administered at different times from the s.c. inoculation of the melanoma cells: (A1 and B1) 1 day before; (A2 and B2) 3 days after; (A3 and B3) 7 days after. The results showed that the best antitumor effect of FPP^®^ was reached with the 200 mg/Kg/mouse dose and when the mice were treated 7 days after the melanoma cells inoculation (*p* < 0.0001). (Figure 2A vs. Figure 2B). Figure 2C shows better the efficacy of both the 200 mg/Kg/mouse and 400 mg/Kg/mouse FPP^®^ doses as administered 7 days after the melanoma cells s.c. inoculation. More in detail at 14 days and at 21 days after B16F10 inoculation the 200 mg/Kg/mouse FPP^®^ treatment induced a 3 folds reduction of the tumor mass as compared to the untreated mice (904 ± 106 mm^3^ vs. 2441 ± 156 mm^3^ respectively, *p* < 0.0001), while the concentration of FPP^®^ of 400 mg/Kg/mouse (group B3) resulted in a tumor reduction of approximately 2-fold (1259 ± 74 mm^3^ vs. 2441 ± 156 mm^3^ respectively, *p* < 0.0001) (Figure 2C). All in all this set of experiments showed that daily FPP^®^ administration by gavage was induced the most significant effect with the dose of 200 mg/Kg/mouse administered 7 days after the melanoma cells s.c. inoculation.

### 2.3. Comparison of the of FPP^®^ Oral Gavage Versus Sublingual on the Growth of Melanoma in C57/BL Mice

On the basis of the above results we wanted to compare different route of FPP^®^ administration in terms of effectiveness on melanoma growth. To this purpose the mice were treated one week after the s.c. inoculation of B16F10 cells with FPP^®^ at a concentration of 200 mg/kg/mouse, every day without interruption until the mice sacrifice by either oral gavage and sublingual administration. Using oral gavage, FPP^®^ was released directly into the stomach of mice through a gastric probe, while the sublingual route was the typical way of taking FPP^®^.

Mice were thus divided into three treatment groups (control, A and B), 6 mice for each group. The control mice were treated exclusively with sterile water, the mice of group A were treated with FPP^®^ by oral gavage and the mice in group B were treated with sublingual FPP^®^.

**Figure 3:**
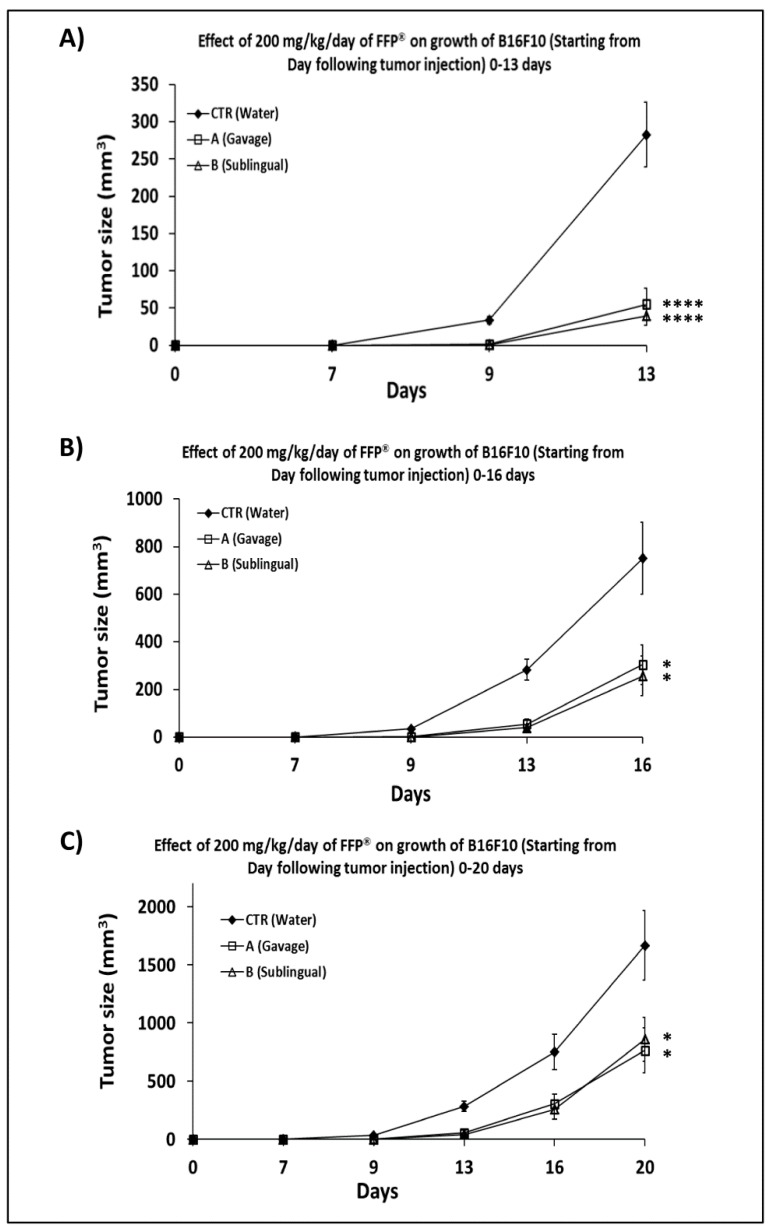
Effect of 200 mg/Kg/day of FPP^®^ on growth of B16F10 in C57/BL mice through oral gavage and sublingual administration. Effect of 200 mg/Kg/day of FPP^®^ on growth of B16F10 in C57/BL mice inoculated with 200,000 B16F10 cells and treated starting 7 days after cell inoculation every day without interruption with FPP^®^ at the concentration of 200 mg/Kg/mouse by oral gavage (group A) and sublingual (group B). Mice were divided into three groups of six animals (control, A, B) and FPP^®^ was performed until animal sacrifice after 20 days from the inoculation of the cells. (**A**) Effect of 200 mg/Kg/day of FPP^®^ on growth of B16F10 in C57/BL mice until 13 days after inoculation of cells. (**B**) Effect of 200 mg/Kg/day of FPP^®^ on growth of B16F10 in C57/BL mice until 16 days after inoculation of cells. (**C**) Effect of 200 mg/Kg/day of FPP^®^ on growth of B16F10 in C57/BL mice until 20 days after inoculation of cells (animal sacrifice). Data are expressed as means ± SE. * *p* < 0.05, **** *p* < 0.0001.

The results in Figure 3 showed that the antitumor effect of FPP^®^ was clearly detectable as early as 13 days after the s.c. inoculation of the cells (*p* < 0.0001). At that time the tumor of the treatment groups were five and seven times smaller (group A = 55 ± 21 mm^3^, *p* < 0.0001; group B = 39 ± 12 mm^3^, *p* < 0.0001), as compared to the control group (283 ± 43 mm^3^) (Figure 3A,C). 

After 16 days from the s.c. inoculation of the melanoma cells the tumor size of the FPP^®^-treated animals were both significantly lower than the control group (751 ± 151 mm^3^): 304 ± 84 in group A (*p* < 0.04) and 256 ± 84 mm^3^ in group B (*p* < 0.02) respectively (Figure 3B,C). At the time of the sacrifice of the mice, 20 days after the s.c. inoculation of B16F10 and 13 days from the beginning of the FPP^®^ treatment, the tumor mass of the untreated mice (1666 ± 298 mm^3^) was 2.2 times than that of group A (764 ± 193 mm^3^, *p* < 0.05) and two times than that of group B (860 ± 188 mm^3^, *p* < 0.05) (Figure 3C). To note we have been obliged to sacrifice all the animals at that time in order to respect established ethical rules. At the post-mortem examination there were no macroscopical evidence of metastasis. 

This set of experiments showed that the daily treatment of mice with FPP^®^ administered by both gavage and sublingual induced a highly significant reduction of tumor growth, slowing down the progression and suggesting therapeutic properties of FPP^®^. Treatment with sublingual FPP^®^ was an ideal, simpler and less stressful route of administration for mice. Our results suggest that the administration of FPP^®^ by “oral gavage” may be stressful for mice, resulting in common complications and usually related to animal resistance to the procedure such as involuntary tracheal administration. Therefore sublingual administration could represent a less aggressive and more suitable alternative.

### 2.4. Combined Preventive and Therapeutic Effects of FPP^®^ on Melanoma Growth

In order to evaluate the combined preventive and therapeutic effects of FPP^®^ on tumor growth, we started the animal treatment by sublingual administration either 21 days before (group A) or 14 days before (group B) or 3 days before (group C) the s.c. melanoma cells inoculation. Briefly, 100000 B16F10 melanoma cells/mouse were inoculated, and the mice were treated with FPP^®^ at the concentration of 200 mg/Kg/mouse each day sublingually without interruption until sacrifice. Group A was treated with FPP^®^ for 44 consecutive days, groups B for 37 days and group C for 26 days respectively.

**Figure 4:**
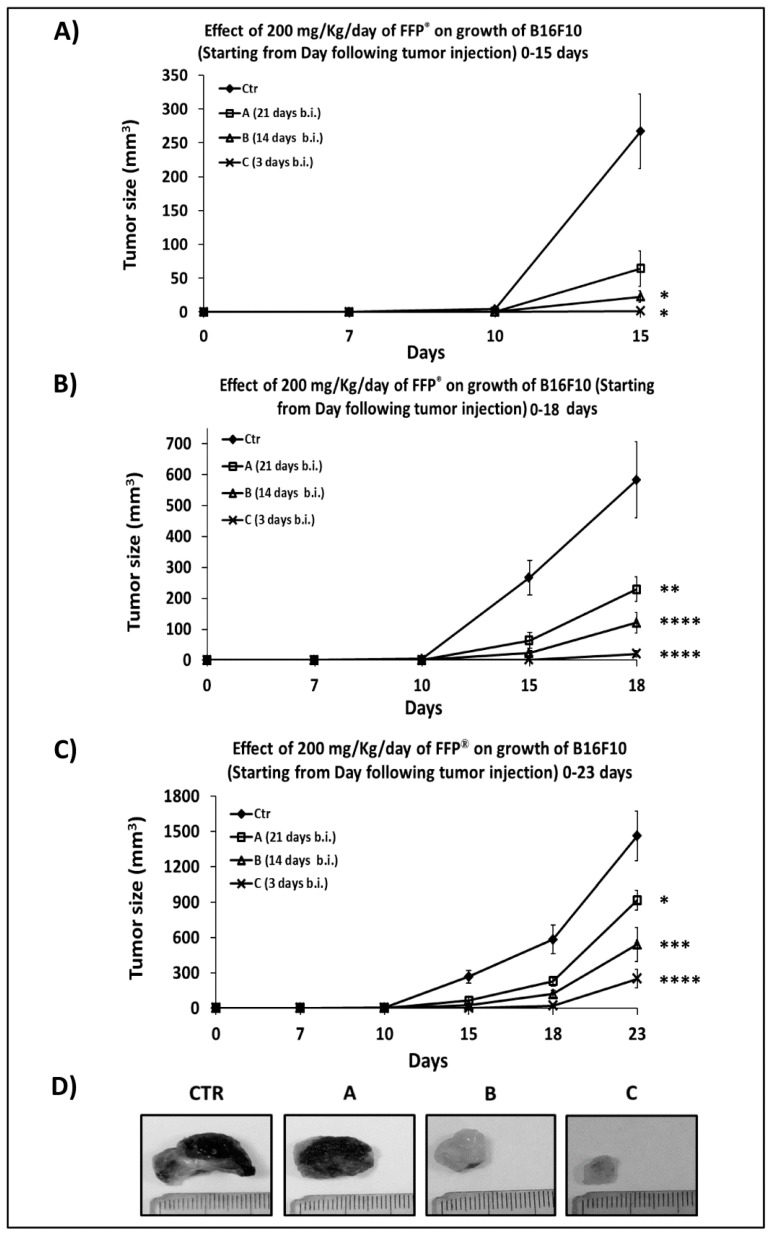
Combined preventive and therapeutic effects of 200 mg/Kg/day of FPP^®^ on growth of B16F10 in C57/BL mice 21 (group A), 14 (group B) and 3 (group C) days before inoculum. Preventive effect of 200 mg/Kg/day of FPP^®^ on growth of B16F10 in C57/BL mice treated 21 days before (group A), 14 days before (group B) and 3 days before (group C) cell inoculation. Mice were divided into 4 groups of 10 animals (control, A, B, C) inoculated with 100,000 B16F10 cells and treated only sublingually with FPP^®^ at the concentration of 200 mg/Kg/mouse every day without interruption until the sacrifice of the animals. (**A**) Effect of 200 mg/Kg/day of FPP^®^ on growth of B16F10 in C57/BL mice until 15 days after inoculation of cells. (**B**) Effect of 200 mg/Kg/day of FPP^®^ on growth of B16F10 in C57/BL mice until 18 days after inoculation of cells. (**C**) Effect of 200 mg/Kg/day of FPP^®^ on growth of B16F10 in C57/BL mice until 23 days after inoculation of cells (animal sacrifice). (**D**) Size and color of melanoma in mice at sacrifice in control, A, B and C groups. Data are expressed as means ± SE. * *p* < 0.05, ** *p* < 0.003, *** *p* < 0.0004, **** *p* < 0.0001.

After 10 days from the inoculation of B16F10, only the tumors of the untreated control mice were measurable actually (4 ± 2 mm^3^). At 15 days after melanoma s.c. inoculation the tumors of the controls reached a size of 267 ± 55 mm^3^: 4-fold greater than those of group A (64 ± 26 mm^3^), 12-fold greater than group B (23 ± 8 mm^3^, *p* < 0.02) and 165-fold greater of group C (1.6 ± 1 mm^3^, *p* < 0.01) (Figure 4A,C). Eighteen days after the inoculation the size of the control (583 ± 123 mm^3^) was 2.5, 5 and 29 times higher as compared to groups A (230 ± 40 mm^3^, *p* < 0.003), B (121 ± 23 mm^3^, *p* < 0.0001) and C (20 ± 10 mm^3^, *p* < 0.0001) respectively (Figure 4B,C). At the time of the sacrifice,(i.e. 23 days after the inoculation of B16F10) the most significant effect induced by sublingual FPP^®^ administration was in group C (FPP^®^ started 3 days before inoculation), with a tumor of size 250 ± 78 mm^3^ (*p* < 0.0001), 6 times lower than the control (1462 ± 213 mm^3^) (Figure 4C). The tumor sizes of mice from groups A (918 ± 85 mm^3^, *p* < 0.05) and B (541 ± 145 mm^3^, *p* < 0.0004) were about two and three times lower than the control (Figure 4C).

This set of results showed that the daily treatment with sublingual FPP^®^ started before the B16F10 cell inoculation had very powerful preventive and therapeutic effects, significantly reducing the growth of melanoma in C57/BL mice (*p* < 0.0001). The greatest effect was achieved when treatment with FPP^®^ started 3 days before the inoculation of B16F10 cells (group C, up to six times lower, *p* < 0.0001). In fact, a prolonged sublingual treatment (groups A and B) was more stressful for mice compared to a treatment for a shorter time (group C). At the time of sacrifice none of the mice had either metastasis or micro-metastases. In addition, the tumor removal at mice sacrifice revealed that the melanomas of mice belonging to groups B and C were completely pigment-free (amelanotic) (Figure 4D), that is considered the first positive response of melanoma to therapy.

### 2.5. FPP^®^ Treatment, Tumor Growth and Blood ROS Levels

In order to evaluate the in vivo mechanism of action of FPP^®^ we analyzed the redox balance in treated vs. untreated mice, To this purpose we measured the total ROS levels in the blood of untreated (control) mice and those treated daily with FPP^®^ starting at either 21 or 14 or 3 days before the inoculation, up to the sacrifice (groups A, B, C). The analysis of the total ROS was done with a colorimetric assay on the red blood cells of the mice taken before the sacrifice.

**Figure 5:**
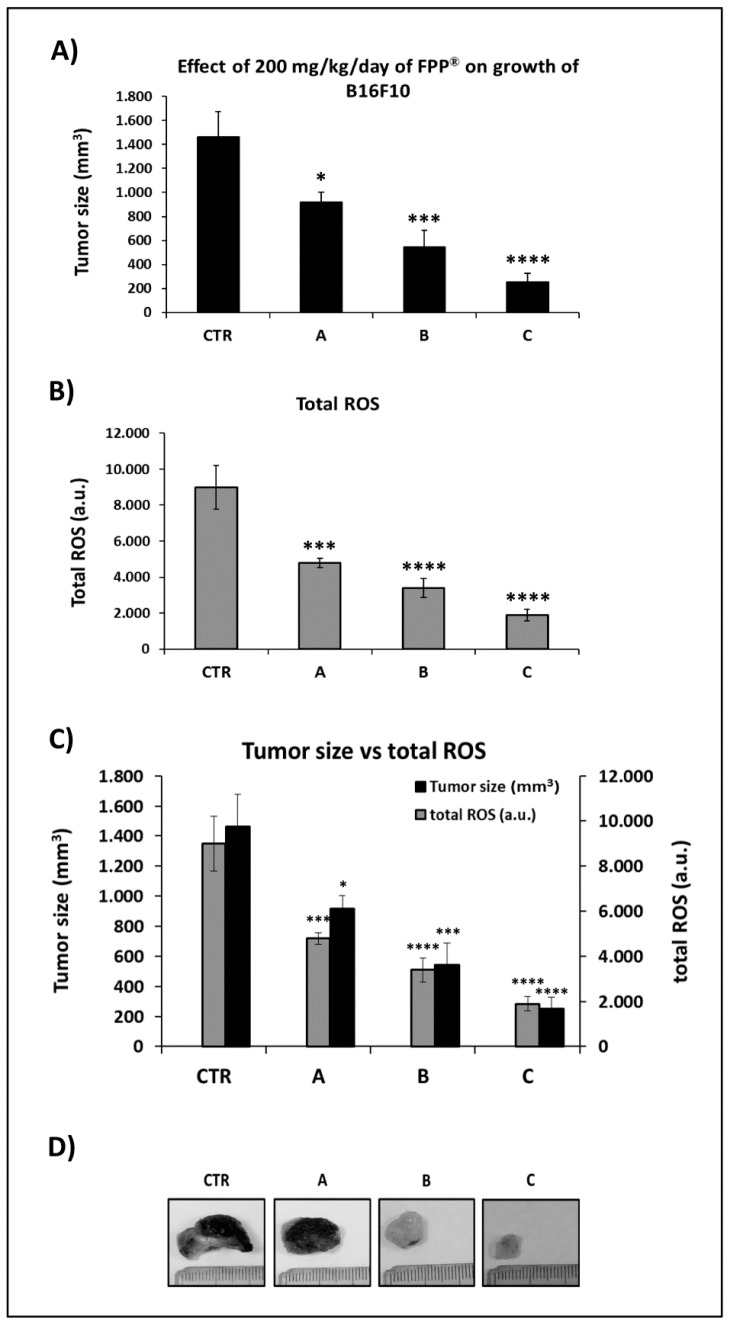
FPP^®^ antioxidant effect by measuring the blood levels of total ROS. Analysis of the total ROS levels (arbitraty units, a. u.) on the blood lymphocytes taken before the sacrifice of the control mice (untreated) and mice treated daily with FPP^®^ starting at 21 days before (group A), 14 days before (group B) and 3 days before (group C) the inoculation up to the sacrifice. Mice were treated only sublingually with FPP^®^ at the concentration of 200 mg/Kg/mouse every day without interruption until the sacrifice of the animals. Analysis of the total ROS levels was performed with a colorimetric assay and measured on a flow cytometer off the 488 nm (blue laser) in the FITC channel. (**A**) Tumor size of B16F10 in untreated mice group (control) and in mice groups treated with 200 mg/Kg/day of FPP^®^ (groups A, B, C). (**B**) Levels of total ROS in control mice group and in mice groups treated with 200 mg/Kg/day of FPP^®^ (groups A, B, C). (**C**) Comparison between tumor size and levels of total ROS in control mice group and in mice groups treated with 200 mg/Kg/day of FPP^®^ (groups A, B, C). (**D**) Size and color of melanoma in mice at sacrifice in control, A, B and C groups. Data are expressed as means ± SE. * *p* < 0.05, *** *p* < 0.001, **** *p* < 0.0001.

The results obtained by the spectrophotometer reading at a wavelength of 488 nm were extremely significant: FPP^®^ induced a highly significant decrease (*p* < 0.0001) in the blood ROS levels, directly related to the size of the tumor (Figure 5). In the untreated mice group (control), in which the tumor growth was much higher than the groups treated with FPP^®^ (A, B, C), higher levels of ROS (8992 ± 1225 a.u.) were measured: the values proven to be either 1.9-, 2.7- or 4.7-fold higher as compared to groups A (4798 ± 259 a.u., *p* < 0.001), B (3391 ± 528 a.u., *p* < 0.0001) and C (1898 ± 327 a.u., *p* < 0.0001) respectively. The lowest total ROS levels were therefore observed in mice in group C, where tumor growth was the lowest actually (Figure 5B,C).

### 2.6. FPP^®^ Treatment, Tumor Growth and Plasmatic Natural Antioxidant (GSH AND SOD-1) Levels

It is known that antioxidant substances, including FPP^®^ may induce a decrease of blood ROS levels by increasing the plasma levels of natural antioxidants, such as free glutathione (GSH) and the enzyme superoxide dismutase-1 (SOD-1). This set of experiments was aimed at establishing the relationship between blood levels of GSH and SOD-1 as compared to the tumor size at the end of FPP treatment.

**Figure 6:**
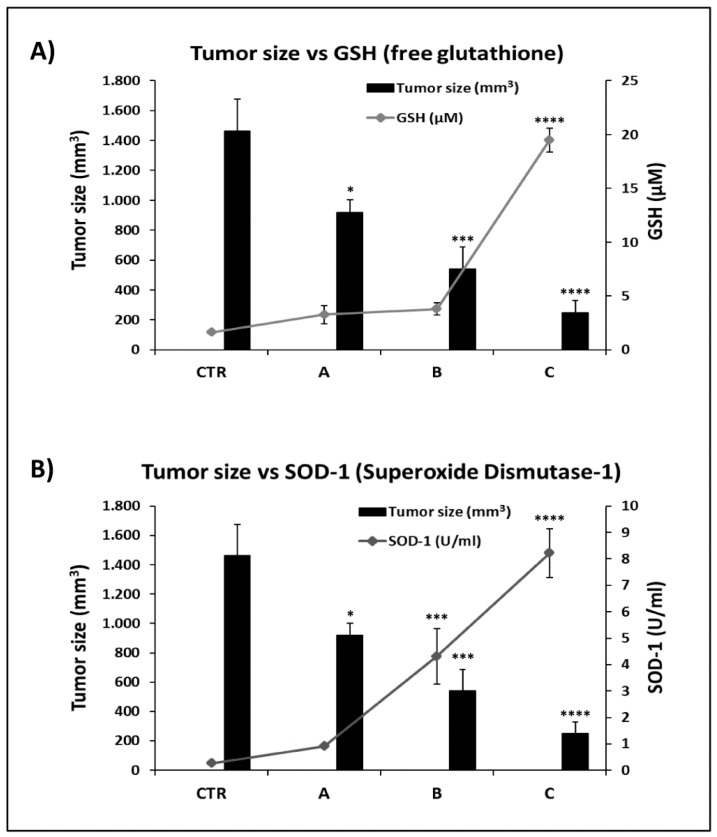
FPP^®^ antioxidant effect by measuring the plasma antioxidant levels (GSH and SOD-1). Analysis of the total plasma antioxidant activity (glutathione GSH and superoxide dismutase SOD-1) taken before the sacrifice of the control mice (untreated) and mice treated daily with FPP^®^ starting at 21 days before (group A), 14 days before (group B) and 3 days before (group C) the inoculation up to the sacrifice. Mice were treated only sublingually with FPP^®^ at the concentration of 200 mg/Kg/mouse every day without interruption until the sacrifice of the animals. (**A**) Comparison between tumor size and GSH activity in control mice group and in mice groups treated with 200 mg/Kg/day of FPP^®^ (groups A, B, C). Analysis of the quantification and detection of GSH activity (µM) was conducted by a colorimetric activity assay the concentration was determined by measuring the absorbance at 405 nm. (**B**) Comparison between tumor size and SOD-1 activity in control mice group and in mice groups treated with 200 mg/Kg/day of FPP^®^ (groups A, B, C). Analysis of the quantification and detection of SOD-1 activity (U/mL) was conducted by a colorimetric activity assay and the absorbance was read at 450 nm. Data are expressed as means ± SE. * *p* < 0.05, *** *p* < 0.001, **** *p* < 0.0001.

The antioxidant analysis was carried out on the plasma obtained from the mice’s blood before their sacrifice and through a colorimetric assay that allowed the reading of the optical density at 405–412 nm for GSH and 450 nm for SOD-1. The results showed the highest GSH levels in group C, where treatment with FPP^®^ started 3 days before the inoculation of B16F10 and continued for 26 consecutive days, with an average value of 19.5 ± 1.1 μM (*p* < 0.0001), higher than 12.1 times compared to the control group (1.6 ± 0.1 μM), 6 and 5.1 times higher as compared to A (3.3 ± 0.8 μM) and B (3.8 ± 0.6 μM) groups, respectively (Figure 6A).

Comparable results were obtained with the SOD-1 levels whose blood values were again significantly higher in group C mice (8.2 ± 0.9 U/mL, *p* < 0.0001 as compared to control group (0.3 ± 0.0 U/mL), group A (0.9 ± 0.1 U/mL) and group B (4.3 ± 1.1 U/mL, *p* < 0.001) (Figure 6B). Notably both SOD-1 and GSH blood levels were always inversely related to the tumor size and the blood ROSA levels of course. 

Therefore FPP^®^ anti-tumor effect was consistent with both the decrease of hydroxyl radicals (the most dangerous among free radicals) levels and the increases of superoxide-dismutase (SOD-1) and glutathione (GSH) antioxidants blood levels. Strongly suggesting that the FPP^®^ antitumor effect passed through a triggering of the natural anti-oxidant mechanisms.

Again the experiments were stopped at the 23th day for ethical reasons having the tumors of control mice reached limit sizes. In conclusion, the best FPP^®^ antitumor effect was obtained with a shorter treatment, indeed sadly the positive effect of FPP^®^ for a longer time was overcome by the stressing daily administration to the mice, inasmuch as also the sublingual administration in experimental animals passed through a non-voluntary procedure being the animals obliged to open the mouth.

## 3. Discussion

A growing body of evidence is demonstrating that tumor growth and progression highly depend on tumor micro-environment that makes hard for either normal or more differentiated cells to survive. In short, the malignant tumor forms and spreads thanks to a progressive selection of cells, already present in our body, which are the most suitable to live in an environment lacking oxygen and nutrients, and last but not least with acidic pH conditions that do not allow the survival of normal cells.

In previous studies we have shown that a promising antitumor approach is the use of antacid substances, such as proton pump inhibitors (PPI) [2,4,5,19,20] or potent buffers [1]. Prevention is playing a key role in the fight against cancer. In this sense, many studies have shown that foods (fruit and vegetable) with a high content of antioxidants such as polyphenols and lycopene are the ideal candidates for cancer prevention [28,29,30,31]. Among these the antioxidant and immunostimulant effect of Fermented Papaya (FPP^®^) was already well known [34,35,36,37], which represents an excellent nutraceutical in many pathologies [36,38,42,43,53,54,55,61] and a promising antitumor agent [50,63]. Before this study the knowledge on the antitumor activity of FPP^®^ was very low or limited to in vitro investigations. 

For this purpose, in this study we used a model of immunocompetent C57/BL mice, inoculated with melanoma B16, to demonstrate the effects of FPP^®^ in either the prevention or treatment of melanoma. We first showed that optimal effect of FPP^®^ treatment on tumor growth was at a dose of 200 mg/Kg per day, treatment starting at 7 days after subcutaneous inoculation of B16F10 melanoma cells. With this experimental approach we obtained a significant reduction of the tumor mass as compared to untreated mice (*p* < 0.0001). As often has been shown in experimental oncology we did not obtain any increase in the antitumor effect of the FPP^®^ treatment while doubling the dose (400 mg/kg/day), comparably to a previous study we performed with a potent buffer [1]. This is called also paradoxical effect and is probably due to the difficulty in setting the weight-established dose in the mice models.

The treatment of mice with FPP^®^ administered through both gavage and sublingual routes always induced a significant reduction of the tumor growth (*p* < 0.03). The antitumor effect of FPP^®^ was evident up to 13 days after melanoma cell inoculation, with only 6 consecutive days of mice treatment. In this case the tumor of the mice treated with FPP^®^ grew up to seven times less than the control (*p* < 0.0001). However, the FPP^®^ anti-tumor effect was clearly dependent on the duration of the treatment being the shortest treatment always the most effective. This result might be explained by the invasive and non-voluntary procedure of forcing the mice to have FPP^®^ orally each day.

Therefore, it is important to consider the methods of administration, as invasive procedures may induce a series of important complications and reactions to the animal that often compromise the effectiveness of the therapy, as it has been clearly shown for the oral gavage route of administration [64,65]. This seems also the case of prolonged sublingual administration that differently to humans receive the sublingual treatment as a non-voluntary procedure. However treatment with sublingual FPP^®^ represented the best route of administration, simpler and less stressful for mice as compared to the oral "gavage" treatment, thus supporting previous studies [66].

Further experiments have shown that FPP^®^ is able to significantly reduce tumor growth. The most significant combined preventive and therapeutic effects were obtained sublingually when FPP^®^ treatment started 3 days before the melanoma B16 inoculation (tumor size up to 6 times lesser than controls, *p* < 0.0001). 

Moreover, at the time of the sacrifice none of the mice treated with FPP^®^ had metastases and the subcutaneous tumors were significantly smaller than untreated mice and were amelanotic. These results suggest that FPP^®^ not only drastically reduced melanoma growth, but also controlled tumor progression by inhibiting metastatic development and inducing the growth of amelanotic tumors. Notably, the amelanotic turning is considered from a clinical point of view the first response of a melanoma to a pharmacological treatment, further supporting a direct effect of FPP^®^ on the progression of a melanoma as aggressive as B16F10.

In order to better understand the mechanism/s underlying the antitumor effect of FPP^®^ and to assess the ability to interfere with the accumulation of oxidizing substances, we measured blood levels of total ROS, an example of oxidant molecule and of GSH and SOD-1, examples of natural anti-oxidant molecules, in untreated mice and mice treated with FPP^®^.

The results showed that the mechanism of action of FPP® was strictly related to the decrease of total ROS levels in the blood and to the increase in the plasma levels of antioxidants, such as free GSH and SOD-1. Small amounts of ROS are tolerated and are inactivated by enzymatic systems such as glutathione and other antioxidants, while an excess of ROS production by the tumor cells induces a pathological condition known as “oxidative stress”. In this condition the enzymatic systems and the intracellular antioxidants are no longer able to counteract the overproduction of free radicals, with consequent cellular damage (involving proteins, lipids, carbohydrates and DNA) that can irreversibly compromise cell function, contributing to tumor progression [9,11,23,24]. In this work we have therefore demonstrated the systemic antioxidant effect mediated by FPP^®^: in fact, treating mice every day with FPP^®^ at the concentration of 200 mg/Kg/mouse, the total ROS levels in the blood decreased (*p* < 0.0001) in a directly proportional way to the size of the tumor, while the levels of plasma GSH and SOD-1 increased (*p* < 0.0001) inversely to the size of the tumor. Our results suggest that the ROS scavenging action of FPP^®^ also passes through the stimulation of the body’s anti-oxidant systems. The accumulation of toxic radicals during the tumor growth may markedly inhibit the immune reaction against tumor or even entirely blocks it. We provide in vivo evidence that an anti-oxidant effect of FPP^®^ may underlay an indirect immune stimulation. So the FPP^®^ immune stimulation may well be mediated by the antioxidant effect [37]. An indirect support to this hypothesis is the in vivo effectiveness of either anti-acidic or buffering treatments on the immune response against melanoma; obtained by counteracting the negative effect of the low micro- environmental pH of tumors on both functionality and viability of anti-tumor immune cells [16,67]. A comparable event may well occur in counteracting the accumulation of oxidants in the microenvironment of tumors. This in turn suggests that the progressive accumulation of oxidant molecules during the tumor growth may well contribute to inhibit the anti-tumor immune response thus participating to the well-known “tumor immune escape”; in turn suggesting that one possible approach to trigger a proper anti-tumor immune response might be to drastically reduce the production and accumulation of oxidants due to the tumor growth, possibly stimulating the body release of an anti-oxidant molecules such as SOD-1 and GSH, as our study strongly suggests.

The results of this study give support to important new view points and hypothesis on cancer. One interesting hypothesis is that of a the implementation of a buffer therapy and a buffer diet in cancer patients [68], and FPP^®^ may well be considered a new element to introduce in an integrative approach in this sense. The other hypothesis is really fascinating and it was proposed from two different angles: one is the microevolutionary development of cancers due to the hostile microenvironment [14]; the other deals with the idea that in cancer development endosymbiotic subsystems have a key role [69]. The progressive accumulation of anti-oxidants into the tumor microenvironment may have a key role in both hypotheses and FPP^®^ may thus represent a new and effective approach to reduce the “hostility” of the tumor microenvironment in possibly tuning a re-differentiation of cancer cells. 

## 4. Materials and Methods

### 4.1. Cell Lines

Metastatic melanoma B16F10 cell line was purchased from ATCC (Milan, Italy). The cells were maintained in RPMI culture medium with 10% of FCS (Invitrogen, Milan, Italy) and antibiotics, at 37 °C in humidified 5% CO_2_. Tumor cells were negative for mycoplasma contamination as routinely tested by PCR (Venor^®^GeM, Minerva Biolabs, Berlin, Germany). 

### 4.2. Immun’Âge^®^ - FPP^®^ (Fermented Papaya Preparation)

FPP^®^ (Immun’Âge^®^) used in our experiments was registered with patent number 6401792 from the Osato Research Institute (Gifu, Japan). Depending on the experiment FPP^®^ was administered by oral gavage or sublingually dissolved in sterile water at concentration of 200 or 400 mg/kg/mouse every day without interruption. Treatment of mice with FPP^®^ was started before or after the B16F10 melanoma cells inoculation and continued until sacrifice of the mice.

### 4.3. In Vivo Studies

All the studies were approved by the ethical committee of the Italian National Institute of Health (Rome) and were conducted in accordance with the current Italian Law (Law 26/2014), authorization n° 792/2017-PR, that regulates experiments in laboratory animals. CB57/BL female mice between 16 and 20 g (4 weeks of age) were purchased from Charles River Laboratories Italia s.r.l., (Calco, Lecco, Italy), and housed in the animal facility of the Italian National Institute of Health. Mice had 10 and 14 h periods of light and darkness respectively, were housed in a different number of animal cages, depending on the experiment, with ad libitum mice chow (Mucedola, Settimo Milanese (MI) I) and water provided through a bottle.

Depending on the experiment between 100,000 and 300,000 B16F10 melanoma cells were subcutaneously injected in the right flank. Tumors were calipered twice a week and mice were weighted once a week. Mice were checked twice a week by a veterinarian responsible for animal welfare monitoring for signs of sufferance such as weight loss, decreased water and food consumption, poor hair coat, decreased activity levels and tumor ulcerations. Endpoints were maximum tumor volume of 2400 mm^3^ accordingly to the guidelines for a correct laboratory practice and signs of poor quality of life.

In the first experiment mice were divided into four groups inoculated with an increasing number of B16F10 cells, starting from 100,000 cells up to 500,000 cells to monitor the growth of melanoma over time. Each group consisted of 5 animals for statistical significance. Mice inoculated with 500,000 B16F10 cells were sacrificed after 17 days from inoculation because the tumor had reached too large a size, while the other mice were sacrificed after 21 days from the inoculation of the cells.

In the second experiment mice were divided into seven groups of five animals (control, A1, A2, A3, B1, B2, B3) inoculated with 300,000 B16F10 cells and treated by oral gavage with FPP^®^ at the concentration of 200 mg/Kg/mouse (groups A1, A2, A3) and 400 mg/Kg/mouse (groups B1, B2, B3), respectively 1 day before the inoculation of the cells (groups A1 and B1), 3 (groups A2 and B2) and 7 (groups A3 and B3) days after the inoculation of the cells. FPP^®^ treatment was performed every day without interruption until animal sacrifice after 21 days from the inoculation of the cells, and the experimental scheme is shown in the Table 1.

In the third experiment mice were divided into three groups of six animals (control, A, B) inoculated with 200,000 B16F10 cells and treated starting 7 days after cell inoculation every day without interruption with FPP^®^ at the concentration of 200 mg/Kg/mouse by oral gavage (group A) and sublingual (group B). FPP^®^ was performed until animal sacrifice after 20 days from the inoculation of the cells, and the experimental scheme is shown in the Table 2.

In the fourth experiment mice were divided into four groups of 10 animals (control, A, B, C) inoculated with 100000 B16F10 cells and treated only sublingually with FPP^®^ at the concentration of 200 mg/Kg/mouse every day without interruption until the sacrifice of the animals. Treatment with FPP^®^ started 21 days before (group A), 14 days before (group B) and 3 days before (group C) cell inoculation. The experimental scheme is shown in the Table 3.

### 4.4. Total Ros Assay

Analysis of the total ROS levels was performed in preparations of the mice red blood cells obtained before the sacrifice. To this purpose a Total Reactive Oxygen Species (ROS) Assay Kit 520 nm (ThermoFisher, Waltham, MA, USA,) was exploited. The cells were resuspended with 100 μL of 1× ROS Assay Stain and incubated for 60 min in a 37°C incubator with 5% CO2. The cells were treated with the desired reagents to induce production of ROS and analyzed on a microplate reader off the 488 nm (blue laser) in the FITC channel. 

### 4.5. SOD Activity Assay

Detection and quantification of superoxide dismutase activity was performed in preparations of the mice plasma obtained before the sacrifice. To this purpose a colorimetric activity assay, The Superoxide Dismutase Activity kit (ThermoFisher). In order to obtain plasma samples, EDTA-treated blood from C57/BL mice injected with murine melanoma were centrifuged at 400*g* for 20 min, plasma was collected and immediately analyzed. The samples were incubated for 20 min at room temperature after the addition of the sample and substrate and chromogenic detection reagent. The optical densities were recorded at 450 nm.

### 4.6. GSH Activity Assay

Detection and quantification of glutathione activity was performed in preparations of the mice plasma before the sacrifice. To this purpose a colorimetric activity assay, Glutathione Colorimetric Detection Kit (Thermo Fisher). In order to obtain plasma samples, EDTA-treated blood from C57/BL mice injected with murine melanoma were centrifuged at 400*g* for 20 min, plasma was collected and immediately analyzed. The samples were incubated for 20 min at room temperature after the addition of the detection reagent and reaction mixture. The optical densities were recorded at 405 nm. 

### 4.7. Statistical Analysis

Results in the text are expressed as means ± standard error (SE), calculated using the GraphPad Prism software San Diego, CA, USA. The statistical analysis was done with one-way ANOVA Bonferroni. Statistical significance was set at *p* < 0.05.

## 5. Conclusions

Early diagnosis and secondary prevention are today the most effective therapeutic strategies in the treatment of melanoma, since the existing therapies are not very effective in favoring appreciable survival rates [70]. The results of our study strongly support the use of FPP^®^ in new anticancer strategies, in particular in the treatment of melanoma both in neoadjuvant regimen and after surgical treatment. FPP^®^ may well implement current anti-tumor strategies but it may also be tested in combination with antacid therapies based on anti-acidic or alkaline substances [1,2,4,5,19,20].

This study represents a milestone pre-clinical evidence showing that a potent anti-oxidant treatment through FPP^®^ may be very promising in both preventing tumors and in implementing current anti-cancer strategies, most of all in combination with chemotherapy that is known to increase the levels of oxidants production and release by our body.

## Figures and Tables

**Table 1:** Experimental scheme of second experiment in C57/Bl mice treated with FPP^®^.

Group	Start of Treatments	Number of Animals	Dosage FPP^®^/Mouse (mg/Kg/day)	Route of Administration
**Control (CTR)**	/	5	sterile water	oral gavage
**A1**	1 day before inoculum	5	200	oral gavage
**A2**	3 days post inoculum	5	200	oral gavage
**A3**	7 days post inoculum	5	200	oral gavage
**B1**	1 day before inoculum	5	400	oral gavage
**B2**	3 days post inoculum	5	400	oral gavage
**B3**	7 days post inoculum	5	400	oral gavage

**Table 2:** Experimental scheme of third experiment in C57/Bl mice treated with FPP^®^.

Group	Start of Treatments	Number of Animals	Dosage FPP^®^/Mouse (mg/Kg/day)	Route of Administration
**Control (CTR)**	/	6	sterile water	oral gavage
**A**	7 days post inoculum	6	200	oral gavage
**B**	7 days post inoculum	6	200	sublingual

**Table 3:** Experimental scheme of fourth experiment in C57/Bl mice treated with FPP^®^.

Group	Start of Treatments	Number of Animals	Dosage FPP^®^/Mouse (mg/Kg/day)	Route of Administration
**Control (CTR)**	/	10	sterile water	sublingual
**A**	21 day before inoculum	10	200	sublingual
**B**	14 days post inoculum	10	200	sublingual
**C**	3 days post inoculum	10	200	sublingual
